# THSD1 Suppresses Autophagy-Mediated Focal Adhesion Turnover by Modulating the FAK-Beclin 1 Pathway

**DOI:** 10.3390/ijms25042139

**Published:** 2024-02-10

**Authors:** Zhen Xu, Jiayi Lu, Song Gao, Yan-Ning Rui

**Affiliations:** 1 Department of Neurosurgery, McGovern Medical School, The University of Texas Health Science Center at Houston, Houston, TX 77030, USA; 2 Department of Pathology, University of Pittsburgh School of Medicine, Pittsburgh, PA 15261, USA

**Keywords:** THSD1, autophagy, focal adhesions, Beclin 1, endothelial cells

## Abstract

Focal adhesions (FAs) play a crucial role in cell spreading and adhesion, and their autophagic degradation is an emerging area of interest. This study investigates the role of Thrombospondin Type 1 Domain-Containing Protein 1 (THSD1) in regulating autophagy and FA stability in brain endothelial cells, shedding light on its potential implications for cerebrovascular diseases. Our research reveals a physical interaction between THSD1 and FAs. Depletion of THSD1 significantly reduces FA numbers, impairing cell spreading and adhesion. The loss of THSD1 also induces autophagy independently of changes in mTOR and AMPK activation, implying that THSD1 primarily governs FA dynamics rather than serving as a global regulator of nutrient and energy status. Mechanistically, THSD1 negatively regulates Beclin 1, a central autophagy regulator, at FAs through interactions with focal adhesion kinase (FAK). THSD1 inactivation diminishes FAK activity and relieves its inhibitory phosphorylation on Beclin 1. This, in turn, promotes the complex formation between Beclin 1 and ATG14, a critical event for the activation of the autophagy cascade. In summary, our findings identify THSD1 as a novel regulator of autophagy that degrades FAs in brain endothelial cells. This underscores the distinctive nature of THSD1-mediated, cargo-directed autophagy and its potential relevance to vascular diseases due to the loss of endothelial FAs. Investigating the underlying mechanisms of THSD1-mediated pathways holds promise for discovering novel therapeutic targets in vascular diseases.

## 1. Introduction

Focal adhesions (FAs) represent subcellular organelles that tether cells to their extracellular matrix. Integrins as transmembrane proteins play a prominent role in FA assembly and function. The extracellular domain of integrin engages various matrix proteins, such as collagens and laminins, while its intracellular region interacts with a diverse array of adaptor proteins such as talin. Consequently, the integrin complex serves as a conduit for sensing extracellular cues and transducing these signals into the cell, ultimately orchestrating cytoskeletal rearrangements for effective cell adhesion [1]. While originally observed in the context of two-dimensional cell culture systems, loss-of-function studies in animal models underscore the critical role of FAs in vascular integrity. For instance, the inactivation of αv or β8 integrin subunits in mice [2,3], or the knockdown of *talin* in zebrafish, leads to cerebral hemorrhage [4]. Furthermore, endothelial-specific depletion of FA kinase (FAK) is implicated in brain hemorrhage [5]. Despite the wealth of research on FA biogenesis, our understanding of the regulatory mechanisms governing FA turnover remains limited.

Emerging evidence indicates that autophagy, an intracellular self-digestion system, contributes to the degradation of FAs. Various stress conditions such as starvation induce autophagy, in which autophagosomes (double-membrane organelles) engulf cargo and subsequently fuse with lysosomes for degradation [6]. In breast cancer cells, autophagy supports cell migration by facilitating the degradation of FA complexes [7,8,9]. Interestingly, distinct molecular mechanisms were uncovered in different cell lines, suggesting that autophagy-mediated FA turnover is context-dependent. Our understanding of how autophagy degrades FAs in primary cells, particularly in primary human brain endothelial cells, remains largely unknown. Bridging this knowledge gap is critical for identifying potential therapeutic targets in the context of cerebrovascular diseases in which endothelial FAs and vascular integrity are often compromised. 

Multiple rare variants in the *THSD1* gene have been identified in patients with intracranial aneurysms, a type of cerebrovascular disease. Loss of THSD1 led to cerebral hemorrhage in both murine and zebrafish models [10], supporting its role in vascular integrity maintenance. Our group has further characterized THSD1 as a novel FA-associated protein, predominantly expressed in endothelial cells while being scarcely detectable in vascular smooth muscle cells [10,11]. However, the precise mechanisms by which THSD1 regulates endothelial FA stability have remained elusive. The purpose of this study is to examine what downstream molecules or signaling pathways contribute to THSD1-mediated FA stability in endothelial cells. Our work reveals that THSD1 functions as a negative regulator of autophagy, and inactivation of THSD1 induces autophagy-dependent FA turnover in primary human brain endothelial cells. Blockade of autophagy restores FA stability, along with FA-associated cell spreading and attachment. Mechanistically, FAK, a master focal adhesion kinase, acts downstream of THSD1. Loss of THSD1 leads to reduced FAK activity, relieving its inhibitory effects on the complex formation between Beclin 1 and ATG14—a crucial event known to activate the autophagy cascade.

## 2. Results

**Figure 1:**
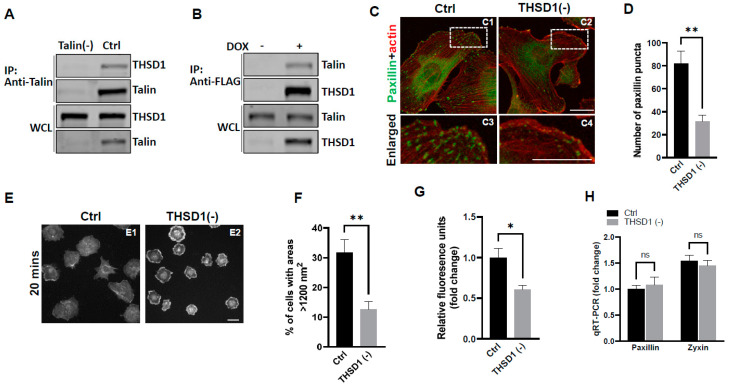
THSD1 interacts with talin and modulates focal adhesion stability. (**A**) Western blot detection of co-immunoprecipitated THSD1 in the talin protein complex from HBMECs treated with siRNAs against *control* (Ctrl, 5 nM) and *talin* (talin (-), 5 nM) for 48 h. To mitigate the potential off-target effects, we utilized two different siRNAs with non-overlapping sequences. Consequently, data from both clones were pooled for statistical analyses, and representative knockdown results were shown. This method will be used for all other genes to be silenced throughout the study unless indicated otherwise. *n* = 3 independent experiments. (**B**) Induction of FLAG-THSD1 in HBMECs with doxycycline (100 ng/mL) for 48 h followed by pull-down using anti-FLAG antibody and Western blot analysis against talin or THSD1. *n* = 3 independent experiments. (**C**,**D**) Representative images of focal adhesions (FAs) immunostained with paxillin (green) and phalloidin (red) in HBMECs treated with siRNAs against *control* (Ctrl, 10 nM) and *THSD1* (THSD1 (-), 10 nM) for 48 h. Areas highlighted by white dashed boxes (C1 and C2) were selected and shown as enlarged pictures at the bottom (C3 and C4). The number of FAs was quantified from at least 12 different fields (20× objective) for each experiment (*n* = 3) and presented in (**D**). Enlarged images were shown in white dashed boxes. (**E**,**F**) Representative images of cell morphologies revealed by phalloidin staining for control or THSD1-deficient HBMECs at 20 min after re-seeding onto chamber slides. Quantitative analysis was performed by counting the number of cells with surface areas exceeding 1200 nm^2^ in each well. At least 9 different fields (10× objective) were randomly recorded for each experiment (*n* = 3). (**G**) Measurement of relative fluorescence units of CyQuant Dye at 480/520 nm from control or THSD1-deficient HBMECs after reseeding onto collagen IV pre-coated 48-well plates. *n* = 3 independent experiments. (**H**) Total mRNA was extracted from both control and THSD1-deficient HBMECs, followed by reverse transcriptase reactions. The mRNA expression levels of paxillin and zyxin were determined through quantitative RT-PCR and subsequently normalized to GAPDH. The fold change was calculated by comparing the mRNA levels in each sample to those in control cells. The statistical comparison was conducted through two-way ANOVA, followed by Bonferroni correction. ns: not significant. * *p* < 0.05; ** *p* < 0.01. ns: not significant. Scale bar: 10 µm.

Our previous investigations have established a physical interaction between THSD1 and talin, a critical component of the integrin complex localized at FA sites [11]. To further confirm this observation, we conducted experiments in primary human brain microvascular endothelial cells (HBMECs), an in vitro model with physiological relevance to cerebrovascular pathology. Endogenous THSD1 was detected in immunoprecipitates pulled down by anti-talin antibodies but was absent in talin-deficient cells (as shown in the first and second lane of Figure 1A). A reciprocal co-immunoprecipitation demonstrated that talin could be pulled down by anti-FLAG antibodies only when THSD1-FLAG was expressed (Figure 1B). These findings support our hypothesis that THSD1 modulates the stability of FAs in brain endothelial cells. To further test this hypothesis, we conducted loss-of-function analyses in HBMECs. Depletion of THSD1 significantly reduced the number of FAs, as indicated by immunofluorescent staining with paxillin antibodies (shown in green in Figure 1C, comparing C3 to C4, and grey to black bars in Figure 1D). Similar results were observed when using zyxin, another marker predominantly identifying mature FAs (Supplemental Figure S1A,B). To minimize potential off-target effects, we conducted *THSD1* knockdown experiments using two different siRNAs with non-overlapping sequences. We observed that FA stability was similarly affected by each siRNA (Supplemental Figure S2A,B). The efficacy of *THSD1* knockdown was verified through Western blot analysis (Supplemental Figure S2C). Furthermore, we observed a significant reduction in the level of F-actin upon THSD1 inactivation (Supplemental Figure S3A,B). This observation is consistent with previous reports indicating a tight association between FAs and stress fibers. The diminished stability of FAs is often associated with impaired cell spreading, as this process relies on interactions between FAs and the extracellular matrix [12]. Our results showed that cell spreading is compromised in THSD1-deficient endothelial cells, with a notable reduction in the percentage of cells exhibiting an area exceeding 1200 nm^2^ within 20 min (Figure 1E, and comparison of grey to black bars in Figure 1F). Later time points showed that stress fibers and FAs form in both control and THSD1-deficient endothelial cells, although the process of cell spreading was delayed due to the inactivation of THSD1 (Supplemental Figure S4). We also observed a significant reduction in cell attachment ability in THSD1-deficient cells, as assessed by the number of remaining cells attached to the pre-coated collagen IV matrix (Figure 1G). Collectively, these data suggest that THSD1 negatively modulates FA stability, cell spreading, and attachment.

To elucidate the mechanisms through which THSD1 downregulates FAs, we performed quantitative polymerase chain reaction (qPCR) analyses on the mRNA level of *paxillin* and *zyxin*. As shown in Figure 1H, there were no discernible changes in the mRNA levels of *paxillin* or *zyxin* following THSD1 depletion. Next, we investigated how THSD1 impacts their protein stability. It is well established that both autophagy and the ubiquitin–proteasome system play crucial roles in regulating protein stability by influencing turnover rates [13,14,15]. To assess the involvement of THSD1 in these degradation pathways, we conducted autophagy flux analyses based on GFP-LC3 puncta formation, along with protein ubiquitination assays as we did previously [16]. LC3 serves as an autophagosomal marker, and GFP-LC3 can be used to monitor autophagic activity. Under conditions of starvation, we observed an increased number of GFP-LC3 puncta (comparison of A2 to A1 in Supplemental Figure S5A, and black bars in S5B). This effect was mitigated upon the knockdown of *ATG5*, the essential gene for autophagy (comparison of A4 to A2 in Supplemental Figure S5A, and grey to black bars in S5B). These data validated the sensitivity and specificity of brain endothelial cells in response to autophagy stimuli.

**Figure 2:**
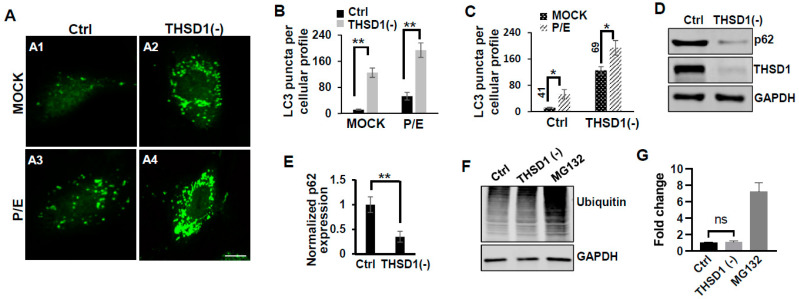
THSD1 inactivation promotes autophagy in endothelial cells. (**A**) Representative images of GFP-LC3 puncta formation in control or THSD1-deficient HBMECs in the absence and presence of pepstatin (50 µg/mL)/E-64D (50 µM) treatment (abbreviated as P/E) for 6 h. Specifically, GFP-LC3 puncta formation in control cells without vs. with P/E treatment (A1 vs. A3) and THSD1-deficient cells without vs. with P/E treatment (A2 vs. A4). (**B**,**C**) Analysis of the effects of THSD1 inactivation on LC3 puncta formation (**B**) and autophagic flux (**C**) in control or THSD1-deficient cells. The number of LC3 puncta per cellular profile was quantified. (**D**) Western blot analysis of p62 protein levels in control or THSD1-deficient HBMECs, with quantification in (**E**). (**F**) Representative images of Western blots against ubiquitinated proteins using the FK1 antibody. GAPDH served as a loading control. (**G**) Fold changes were calculated after normalization to the total ubiquitinated protein level in the control sample (n = 3 independent experiments). * *p* < 0.05; ** *p* < 0.01. ns: not significant. Scale bar: 10 µm.

In this reporter cell line, knockdown of THSD1 resulted in an increased number of green puncta (comparison of A2 to A1 in Figure 2A and grey to black bars in 2B). This phenotype may result from increased autophagosome biogenesis or compromised autophagic degradation that is otherwise indicative of reduced autophagic flux. To distinguish between these possibilities, we conducted standard flux analyses using pepstatin/E-64D (P/E), two protease inhibitors commonly used to prevent cargo degradation within autolysosomes [17]. Our results indicated that autophagic degradation remained unimpaired since P/E treatment further increases the number of GFP-LC3 puncta in both control and THSD1-deficient cells (comparison of A3 to A1, and A4 to A2 in Figure 2A, quantification depicted in Figure 2B,C). We also performed a cargo-based endpoint autophagy assay and found that the level of p62, a bona fide autophagy substrate, was significantly reduced in THSD1-deficient cells (Figure 2D,E). These data support the notion that THSD1 exerts a negative regulatory influence on autophagy in endothelial cells. In contrast, the overall level of protein ubiquitination remained unaltered following *THSD1* knockdown, while the treatment of MG132, the proteasome inhibitor, dramatically induced the accumulation of ubiquitinated substrates (Figure 2F,G).

**Figure 3:**
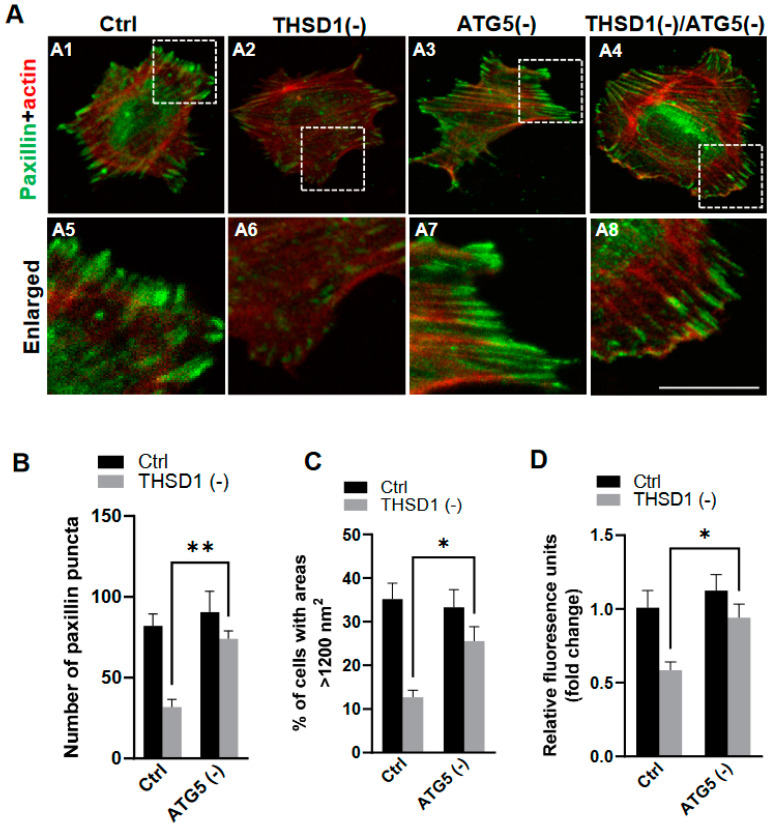
Autophagy inhibition rescues focal adhesion stability. (**A**) Representative images of FAs immunostained with paxillin (green) and actin (red) in control or THSD1-deficient HBMECs treated with siRNAs against *control* (5 nM) or *ATG5* (5 nM). Specifically, FAs were examined in control cells (A1), THSD1-deficient cells (A2), ATG5-deficient cells (A3) and cells that deficient in both THSD1 and ATG5 (A4). Representative areas were highlighted by white dashed boxes and accordingly selected as enlarged images at the bottom (A5–A8, respectively). (**B**) Quantification of the number of paxillin puncta per cellular profile from at least 12 different fields (20× objective) for each experiment (*n* = 3). (**C**,**D**) Evaluation of cell spreading (**C**) and attachment (**D**) in control or THSD1-deficient HBMECs in the absence and presence of *ATG5* siRNA (5 nM) treatment for 48 h. Data were analyzed by two-way ANOVA followed by Bonferroni correction. * *p* < 0.05; ** *p* < 0.01. Scale bar: 10 µm.

To investigate whether THSD1-mediated autophagy contributes to FA stability, we performed an FA assay by further knockdown of *ATG5* in both control and THSD1-deficient cells. *ATG5* knockdown effectively rescued the defective FAs in THSD1-deficient cells (comparison of the number of green puncta in A4 to A2, or A8 to A6 in enlarged pictures, as well as the grey bars in Figure 3B). We noticed that both the number and size of FAs were reduced in THSD1-deficient cells. Moreover, knockdown of *ATG5* restored cellular spreading efficiency and attachment ability in THSD1-deficient cells (comparison of grey bars in Figure 3C,D). The specificity and effectiveness of *ATG5* knockdown were confirmed using two independent siRNAs, as assessed by FA stability assay and total protein level, respectively (Supplemental Figure S6). To investigate whether THSD1 inactivation influences the level of ATG5, or vice versa, we conducted Western blot analysis on total lysates from HBMECs deficient in either protein (Supplemental Figure S7). Our data suggest that the rescue effects observed with ATG5 inactivation are not directly attributed to the upregulation of THSD1 but rather are likely a result of preventing autophagy-mediated degradation. In contrast, proteasome system inhibition via knockdown of the essential proteasome subunit 2 (*PSMB2*) failed to restore these phenotypes (Supplemental Figure S8), implying a lack of involvement of the ubiquitin proteasome system in THSD1-regulated FA stability.

**Figure 4:**
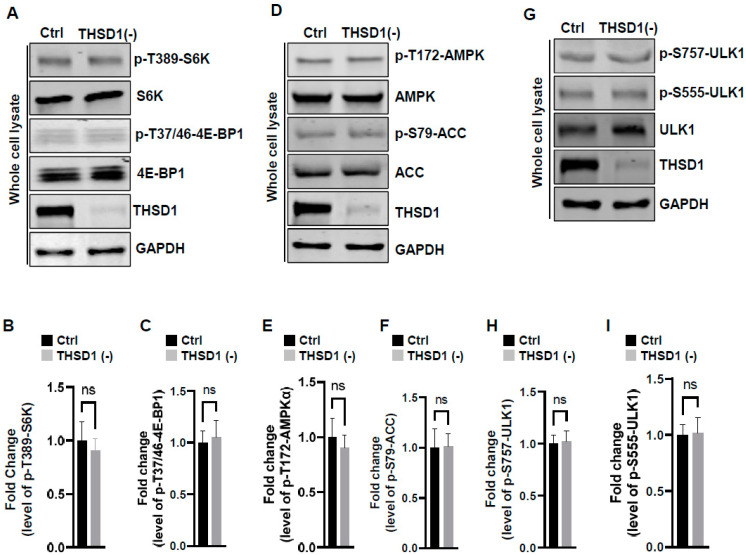
THSD1-mediated autophagy is independent of mTOR or AMPK pathway. (**A**–**C**) Western blot analysis of whole cell lysates (WCL) from control or THSD1-deficient HBMECs using antibodies against p-S6K-T389, S6K, p-4E-BP1-37/46, and 4E-BP1. GAPDH served as a loading control. Quantification of p-S6K-T389 (**B**) and p-4E-BP1-T37/46 (**C**) levels was analyzed by Student’s *t*-test (*n* = 3 independent experiments). (**D**–**I**) Representative Western blots of WCL from control or THSD1-deficient HBMECs for analyzing AMPK signaling (**D**–**F**) or ULK1 activation (**G**–**I**). Quantification of p-AMPKα-T172 (**E**), p-ACC-S79 (**F**), p-ULK1-S757, and p-ULK1-S555 was analyzed by Student’s *t*-test (*n* = 3 independent experiments). ns: not significant.

To examine the mechanisms underlying THSD1-mediated autophagy activation, we conducted a series of signaling pathway analyses. The prior literature suggests that downregulation of mTOR kinase, a central nutrient sensor, or upregulation of AMPK, a principal energy sensor, can potentiate autophagy under conditions of starvation [18,19]. To assess mTOR activity, we conducted immunoblotting to assess the phosphorylated levels of S6K and 4E-BP1, two canonical substrates of mTOR kinase. Knockdown of THSD1 had little effect on either of these readouts in terms of p-T389-S6K and p-T37/46-4E-BP1 (Figure 4A–C). AMPK activation was also not affected, as evidenced by the levels of p-T172-AMPK and p-S79-ACC (Figure 4D–F). ULK1, downstream of mTOR and AMPK, undergoes phosphorylation in response to autophagy induction [20,21]. We assessed the levels of phosphorylated ULK1 at Ser 757 (a target of mTOR) and at Ser 555 (a target of AMPK) but did not observe any discernible changes (Figure 4G–I). These data suggest that THSD1-mediated autophagy operates independently of both mTOR and AMPK.

**Figure 5:**
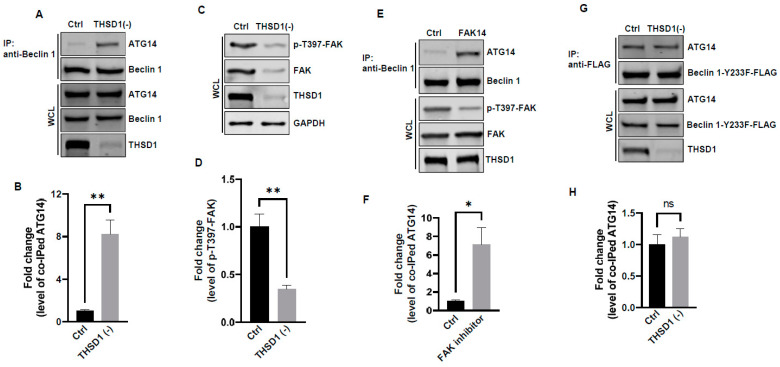
FAK and Beclin 1 are required for THSD1-mediated autophagy. (**A**,**B**) Western blot analysis of immunoprecipitated Beclin 1 protein complexes from control or THSD1-deficient HBMECs using the ATG14 antibody, with quantification in (**B**) (*n* = 3). (**C**,**D**) Western blot analysis of p-FAK-T397 levels in control and THSD1-deficient HBMECs, with quantification in (**D**) (*n* = 3 independent experiments). (**E**,**F**) Immunoprecipitation of Beclin 1 protein complex from HBMECs treated with water or FAK inhibitor 14 (FAK14, 2 µM) for 1 h. Western blot detection of co-immunoprecipitated ATG14 (**E**) and quantification in (**F**) (*n* = 3 independent experiments). (**G**,**H**) Pull-down of Beclin 1-Y233F-FLAG from WCL in control or THSD1-deficient HBMECs, with Western blot detection of co-immunoprecipitated ATG4 (**G**) and quantification in (**H**) (*n* = 3 independent experiments). * *p* < 0.05; ** *p* < 0.01. ns: not significant.

In light of these results, ULK1 was ruled out as a contributor to THSD1-mediated autophagy. We explored the possibility of other downstream molecules or signaling pathways being activated following THSD1 depletion. Beclin 1, an essential and sufficient component for autophagy induction, is positioned downstream of ULK1 within the autophagy pathway. In response to autophagic stimuli, Beclin 1 forms a complex with ATG14 to activate autophagy [22,23]. We observed a significant enhancement in the physical interaction between Beclin 1 and ATG14 in THSD1-deficient endothelial cells (Figure 5A,B). This suggests that THSD1 negatively regulates the formation of the Beclin–ATG14 complex. Focal adhesion kinase (FAK) has been reported to phosphorylate Beclin 1 at Y233, a modification that disrupts the binding of Beclin 1 to ATG14 [24]. Our data demonstrated that *THSD1* knockdown reduced the levels of both FAK and phosphorylated FAK at T397 (Figure 5C,D). Therefore, we propose that THSD1 modulates the FAK/Beclin 1 signaling in autophagy. To examine the role of FAK in Beclin 1–ATG14 protein complex formation, we treated HBMECs with FAK inhibitor 14, a compound that specifically inhibits FAK kinase activity. The interaction between Beclin 1 and ATG14 was significantly enhanced by the treatment (Figure 5E,F). In contrast, a Beclin 1 mutant (Beclin 1-Y233F) that is resistant to FAK phosphorylation strongly bound to ATG14 in both control and THSD1-deficient endothelial cells (Figure 5G,H). Overexpression of this kinase dead mutant reduced the number of FAs, while the phosphor-mimetic mutant Beclin 1 Y233E has little effect (Supplemental Figure S9). A reduced amount of paxillin was also observed in THSD1-deficient endothelial cells (Supplemental Figure S10). These findings suggest that the loss of THSD1 reduces FAK activity, thereby relieving its inhibitory influence on Beclin 1–ATG14 complex formation and consequently leading to the activation of autophagy and turnover of FAs. 

**Figure 6:**
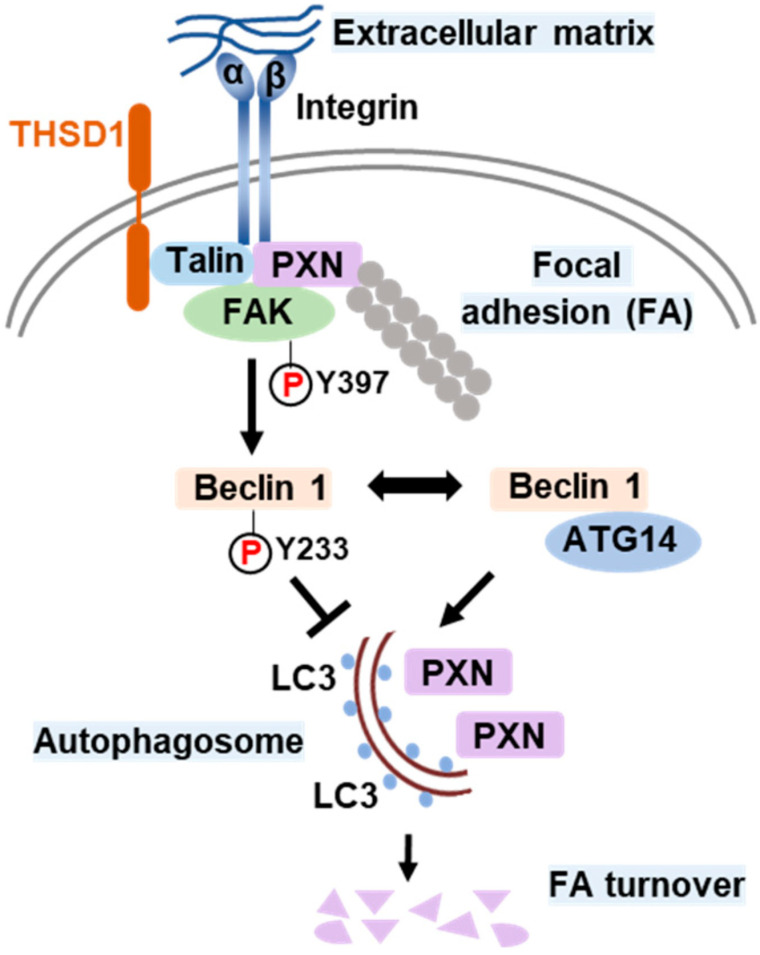
Schematic model of THSD1-mediated autophagy in focal adhesion stability. Focal adhesions (FAs) are composed of integrin α/β transmembrane proteins that link extracellular matrix components like collagens to intracellular adaptors such as talin and paxillin (PXN). The actin cytoskeleton, represented by aligned round grey dots, is tethered to integrins through PXN. Focal adhesion kinase (FAK) plays a central role in focal adhesion regulation, binding to both PXN and THSD1. Under normal conditions, THSD1 interacts with FAK, enhancing its kinase activity, resulting in the phosphorylation of Beclin 1 at tyrosine 233. This phosphorylation event inhibits the binding of ATG14 to Beclin 1, a critical step in autophagy activation. When THSD1 is inactivated, FAK kinase activity is reduced, alleviating its negative control over the formation of the Beclin–ATG14 complex. This, in turn, promotes Beclin 1-dependent autophagosome formation, where LC3 proteins are incorporated on both sides of the phagophore. These autophagosomes can sequester paxillin and other focal adhesion proteins, leading to autophagy-mediated focal adhesion turnover. P: phosphorylation.

Based on all the above results, we propose a model that delineates the role of THSD1 in the regulation of autophagy and FAs (Figure 6). In endothelial cells, FAs comprise the integrin complex, which engages with the extracellular matrix on one end and interacts with adaptor proteins, such as talin and paxillin, on the other. The subtype of each integrin subunit is not specified since talin binds to different β integrin tails. THSD1 localizes at FAs by physically interacting with talin. FAK is dynamically recruited to FAs through its interaction with paxillin. Under physiological conditions, THSD1 preserves FAK activity at FAs. Active FAK phosphorylates Beclin 1 at Y233, which prevents the binding of Beclin 1 to ATG14 and consequently inhibits autophagy. However, under pathological conditions, the loss of THSD1 destabilizes FAK and relieves its inhibitory effects on Bec1in1–ATG14 complex formation. Consequently, Beclin 1-mediated autophagy degrades FAs, which impairs cell spreading and attachment and may ultimately compromise endothelial integrity and promote the development of vascular diseases.

## 3. Discussion

**THSD1 and FA-phagy**: Our work supports THSD1 as a hitherto unrecognized player in the regulation of autophagy within endothelial cells. Our investigation revealed that inactivation of THSD1 did not lead to changes in mTOR and AMPK activation, as depicted in Figure 4. These findings suggest that the primary function of THSD1 may be closely tied to FA dynamics rather than serving as a global regulator of nutrient and energy status. This localized activation of autophagy aligns with the concept of “quality control autophagy”, an emerging paradigm emphasizing the selective type of autophagy that targets distinct cellular organelles, such as FAs, to ensure cellular homeostasis. The phenomenon of autophagy-mediated FA degradation is often referred to as “FA-phagy”, which adds an important layer to our understanding of FA dynamics [13]. Our results support that the activation of FA-phagy is a response to cellular stress arising from impaired cell adhesion, rather than being driven by nutrient or energy depletion.

FAs play a central role in various cellular functions, encompassing cell adhesion, migration, and tissue organization. The disruption of FA stability upon the loss of THSD1 serves as a trigger for localized autophagy at these sites, representing a mechanism of cellular adaptation. Intriguingly, previous studies have documented that Beclin 1 can be localized at the endoplasmic reticulum, and this compartmentalized Beclin 1 supports autophagosome biogenesis even in the absence of ULK1 and ULK2 kinases [25]. This observation is in line with our results, indicating that THSD1-mediated autophagy operates independently of ULK1 activation. Notably, it has been reported that FAK directly interacts with Beclin 1, suggesting that Beclin 1 may also be compartmentalized at FAs [24]. In the future, it would be highly valuable to investigate whether THSD1 facilitates the localization of Beclin 1 at FAs and potentially suppresses its activity by promoting the interaction between FAK and Beclin 1. Such protein complex formation may play a pivotal role in shaping the precise regulation of FA-phagy and its impact on cellular adhesion processes. This intriguing possibility highlights the multifaceted nature of THSD1 in autophagy and FA dynamics, warranting further exploration in subsequent studies.

**Potential link between THSD1-mediated FA-phagy and vascular diseases**: We previously reported that THSD1 is essential for vascular integrity in both zebrafish and mouse models. Our data for the first time connected THSD1-regulated, autophagy-mediated FA stability in normal cells to vascular integrity under physiological conditions. Interestingly, multiple harmful rare variants in the *THSD1* gene have been identified in patients with intracranial aneurysms—a cerebrovascular disorder characterized by the weakening of blood vessel walls. These THSD1 rare variants exhibit varying degrees of impairment in their ability to interact with talin [10,11]. By establishing THSD1 as a regulator of autophagy and FA stability (Figure 1, Figure 2 and Figure 3), our work opens a window of opportunity for investigating the contribution of THSD1-mediated pathways to the pathophysiology of vascular diseases, particularly those involving compromised endothelial integrity. It will be intriguing to determine whether THSD1 rare variants lack the capacity to suppress autophagy. Further investigations into the mechanisms by which THSD1 influences these processes may provide valuable insights into the development and progression of vascular diseases such as intracranial aneurysms, potentially unveiling novel therapeutic targets for intervention.

In conclusion, our study highlights the novel role of THSD1 in modulating autophagy that degrades FAs within endothelial cells. We elucidate a novel mechanism involving the regulation of the Beclin 1–ATG14 complex and emphasize that THSD1 as an FA-associated protein regulates cargo-initiated selective autophagy. These findings broaden our understanding of autophagy regulation and its implications in vascular integrity, paving the way for future investigations into the role of FA-phagy in vascular diseases and related pathologies.

## 4. Materials and Methods

### 4.1. Plasmids

The pLVX-TetOne-Puro vector was obtained from Takara Inc and subsequently modified to incorporate a new polylinker with several 8-cutter sites, including PacI and NotI, to facilitate cloning. This customized vector was referred to as pLTO-PANBR. Full-length *THSD1-FLAG*, *Beclin 1-Y233F-FLAG*, and *Beclin 1-Y233E-FLAG* were cloned into the pLTO-PANBR vector using the PacI and NotI restriction sites through standard PCR protocols. The constructs were verified by Sanger sequencing as previously reported [16]. The pBabepuro-GFP-LC3 plasmid was sourced from Addgene (#22405, Watertown, MA, USA).

### 4.2. siRNAs

All stealth small interfering RNAs (siRNAs) were purchased from ThermoFisher Scientific (Waltham, MA, USA) and included siRNAs targeting *talin* (HSS110804 and HSS186350), *THSD1* (HSS148179 and HSS148180), *ATG5* (HSS114103 and HSS114104), and *PSMB2* (HSS108676 and HSS108677). The effectiveness of each siRNA in knockdown experiments was validated through Western blotting. For all the knockdown experiments, we did not find differences between the two siRNAs against the same gene. Consequently, data from both clones were pooled for statistical analyses and representative gene knockdown results were shown. Knockdown of *talin*, *THSD1*, *ATG5*, and *PSMB2* was accordingly labeled as talin (-), THSD1 (-), ATG5 (-), and PMSB2 (-). 

### 4.3. Antibodies

S6K (9202), p-S6K-T389 (9205), 4E-BP1 (9452), p-4E-BP1-T37/46 (9459), AMPK-α (2532), p-AMPKα-T172 (2535), Acetyl-CoA Carboxylase/ACC (3662), p-ACC-S79 (3661), ULK1 (8054), p-ULK1-S757 (14202), p-ULK1-S555 (5869), ATG14 (5504), Beclin 1 (3495), FAK (3285), p-FAK-T397 (3283), and p62 (8025) were obtained from Cell Signaling Technology, Danvers, MA, USA. THSD1 (NBP1-86930, Novus Biological, Centennial, CO, USA), paxillin (#612405, BD Biosciences, Franklin Lakes, NJ, USA), zyxin (MAB2610, MilliporeSigma, Burlington, MA, USA), ubiquitin (04-262, MilliporeSigma), GAPDH (sc-32233, Santa Cruz Technology, Dallas, TX, USA), and anti-FLAG antibody (F1804, Sigma-Aldrich, St. Louis, MO, USA) were also used.

### 4.4. Cell Culture

Human brain microvascular endothelial cells (HBMECs), obtained from Cell Systems (ACBRI 376, Kirkland, WA, USA), were cultured in endothelial cell medium (#1001, ScienCell Research Laboratories, Carlsbad, CA, USA). Inducible gene expression in HBMECs was achieved through lentiviral infection. Lentiviruses were generated in HEK293T cells with the assistance of two other plasmids, pMD2.G (#12259, Addgene) and psPAX2 (#12260, Addgene). For siRNA-mediated knockdown experiments, siRNAs were introduced into HBMECs using standard electroporation techniques (Neon Transfection System, Invitrogen, Carlsbad, CA, USA). Autophagy was induced by treating HBMECs with Earle’s Balanced Salt Solution (EBSS) for 2 h, followed by LC3 puncta or p62 turnover assays. All chemicals, including pepstatin/E-64D, MG132, and FAK inhibitor 14, were purchased from Cayman Chemical.

### 4.5. Quantitative RT-PCR

Total mRNA was isolated from HBMECs using TRIzol (15596026, ThermoFisher Scientific) and quantified following the Fast SYBR green protocol. The primer sequences are provided below.

Paxillin: 5′-CTGATGGCTTCGCTGTCGGATT/5′-GCTTGTTCAGGTCAGACTGCAG.

Zyxin: 5′-TTCCACATCGCCTGCTTCACCT/5′-CGCAGGTGTTACACTTCTCCAG.

### 4.6. Western Blot

HBMECs were subjected to siRNA or compound treatments in 60 mm dishes and lysed in Triton X-100 lysis buffer, as previously described [26]. The lysates were sonicated briefly and centrifuged at 13,000 rpm for 30 min at 4 °C. Total cell lysates were supplemented with 2× SDS sample buffer and then subjected to SDS-PAGE analysis. The proteins were subsequently transferred onto nitrocellulose membranes using a Bio-Rad mini transfer apparatus. Following this, the membranes were blocked with 5% non-fat milk, and primary and secondary antibodies were applied at dilutions of 1:1000 and 1:10,000, respectively. The Odyssey Imager system (LI-COR Biosciences, Lincoln, NE, USA) was used to detect fluorescence signals.

### 4.7. Immunoprecipitation (IP)

HBMECs treated with siRNAs or compounds in 60 mm dishes were lysed in an IP-lysis buffer, as previously described [26]. The cell lysate was briefly sonicated and then centrifuged at 13,000 rpm for 30 min at 4 °C. Endogenous or exogenous proteins were immunoprecipitated from the cell lysate using either anti-THSD1 or anti-FLAG antibodies in combination with protein A/G Plus agarose beads (Santa Cruz Biotechnology, sc-2003). The immunoprecipitates and whole cell lysates (WCLs) were subjected to standard Western blotting.

### 4.8. Immunostaining

HBMECs treated with siRNAs were reseeded onto 8-well chamber slides at appropriate cell concentrations. After fixation with 4% paraformaldehyde and permeabilization with Triton X-100, the cells were blocked with 10% normal goat serum for 1 h at room temperature. Next, the cells were incubated with primary antibodies at 4 °C overnight and subsequently stained with Alexa-594 or Alexa-488 conjugated secondary antibodies (Invitrogen). The stained samples were mounted in Prolong Gold solution (Invitrogen), and images were captured using a Leica TCS SP5 confocal microscope.

### 4.9. Cell Spreading Assay

HBMECs treated with various siRNAs were trypsinized and then inactivated by endothelial cell medium before resuspension in 1× PBS. The cells were immediately reseeded onto 8-well chamber slides pre-coated with Collagen-IV. After 20 min, the cells were fixed with 4% paraformaldehyde in PBS, and the overall morphology was highlighted by immunostaining against the cytoskeleton protein actin using Alexa 594-phalloidin, as previously reported [11]. Widefield images were acquired using a Leica DM4 microscope with a cMOS camera, and at least 9–12 different fields were chosen from each chamber. Cell areas were measured using ImageJ, and data were analyzed by Student’s *t*-test.

### 4.10. Cell Adhesion Assay

To evaluate cell adhesion, HBMECs subjected to various siRNA treatments were assessed using the CytoSelect Cell Adhesion Assay Kit (MBS168514, Cell Biolabs, San Diego, CA, USA). Cells were trypsinized and resuspended in serum-free endothelial cell medium before adding 150 µL of the cell suspension to each well of a 96-well plate pre-coated with collagen IV. Following a 1 h incubation in a cell culture incubator, the culture medium was removed, and the adherent cells were washed three times with 1× PBS. The remaining adherent cells on the well bottoms were lysed using the 1× Lysis Buffer/CyQuant Dye provided in the kit. Subsequently, a 150 µL aliquot of the lysate was transferred to a 96-well plate, and fluorescence signals were measured using a plate reader at an excitation wavelength of 480 nm and an emission wavelength of 520 nm.

### 4.11. LC3 Puncta Formation and p62 Turnover Assays

The LC3 puncta formation and p62 turnover assays were conducted following established protocols [16]. To assess LC3 puncta formation, cells were initially fixed using 4% paraformaldehyde, followed by permeabilization with 50 µg/mL digitonin. The primary antibody utilized was anti-GFP (GFP-1020, Aves Labs, Davis, CA, USA), and the secondary antibody was Alexa-488 anti-chicken IgG (Invitrogen). For each assay condition, puncta profile data were calculated by averaging the total number of GFP-LC3-positive puncta per cell, drawing from approximately 30–50 representative cells. In the p62 turnover assay, HBMECs treated with various siRNAs for 48 h were harvested in Triton lysis buffer. Western blot analysis was then carried out to determine the protein levels of p62.

### 4.12. Statistics Analysis

Statistical analysis was conducted using GraphPad Prism 6. *p*-values were calculated using the Student’s *t*-test or one- or two-way ANOVA, with Bonferroni correction applied for multiple comparison tests between selected pairs. Data are presented as mean ± standard error of the mean (s.e.m.). A significance level of *p* < 0.05 was considered statistically significant.

## Data Availability

The data that support the findings of this study are available from the corresponding author upon reasonable request.

## Supplementary Materials

The following supporting information can be downloaded at: https://www.mdpi.com/article/10.3390/ijms25042139/s1.
