# Supplementary material for: THSD1 Suppresses Autophagy-Mediated Focal Adhesion Turnover by Modulating the FAK-Beclin 1 Pathway
Source: Int J Mol Sci. 2024 Feb 10;25(4):2139. doi: 10.3390/ijms25042139 (PMC10889294; doi:10.3390/ijms25042139)
Supplement: Supplementary file 1 [file ijms-25-02139-s001.zip › ijms-2786695-supplementary.pdf]

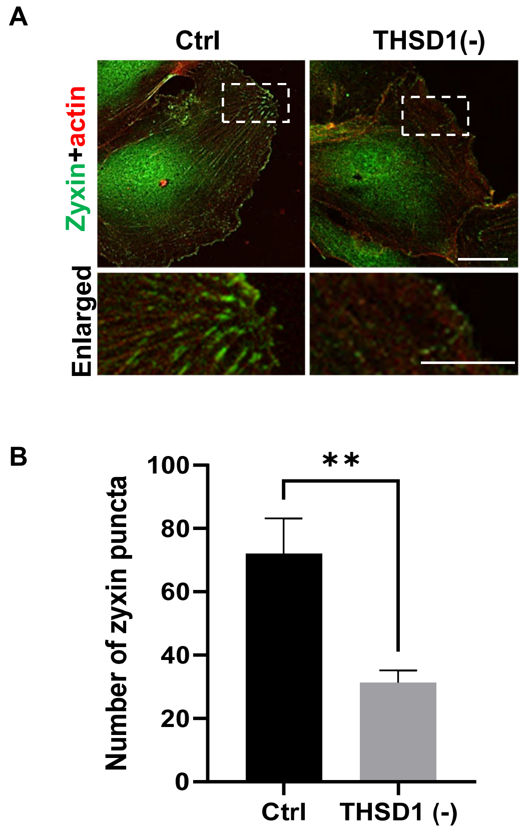

Supplemental Figure S1: Inactivation of THSD1 reduces the number of mature focal adhesions

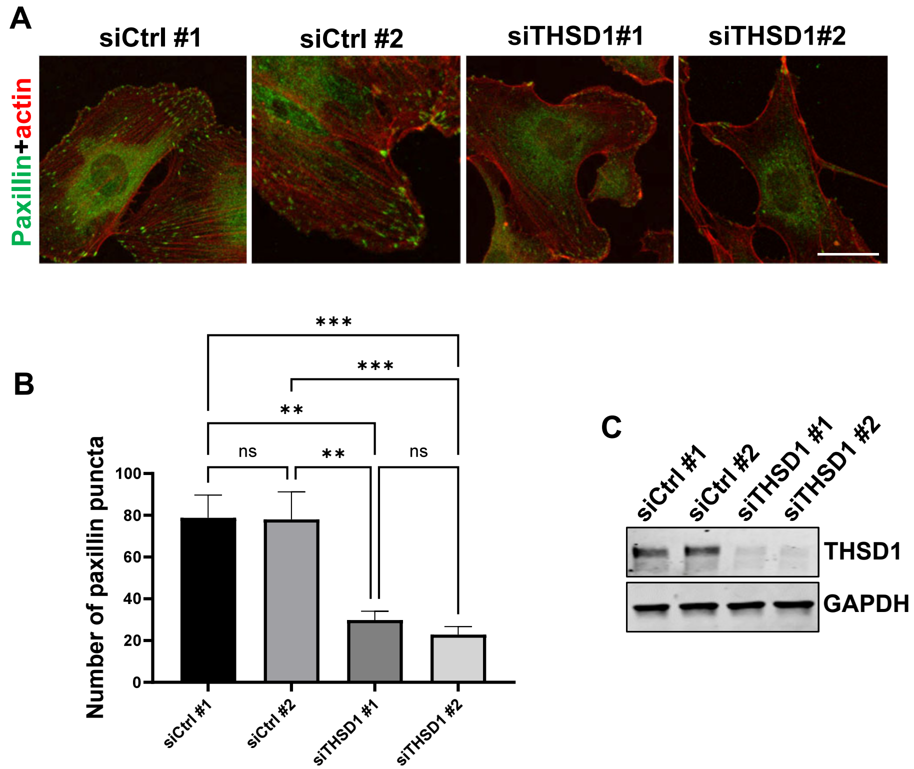

Supplemental Figure S2: Two different siRNAs against THSD1 have similar effects on FA stability

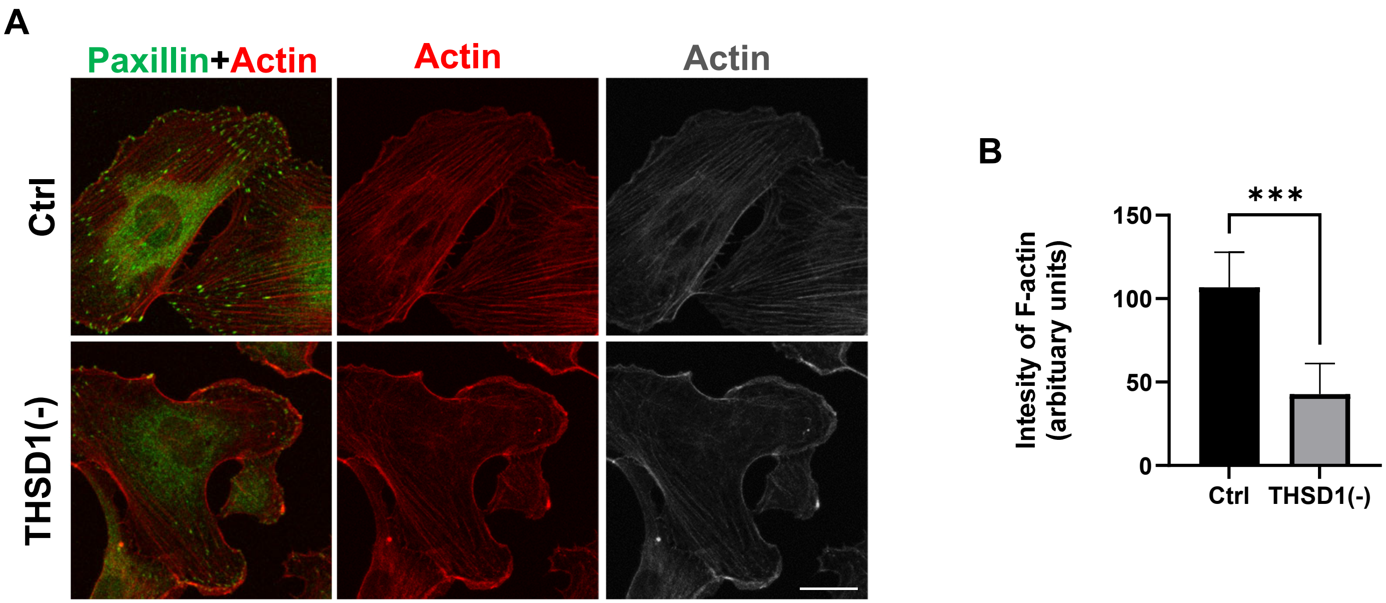

Supplemental Figure S3: THSD1 inactivation reduces the level of F-actin

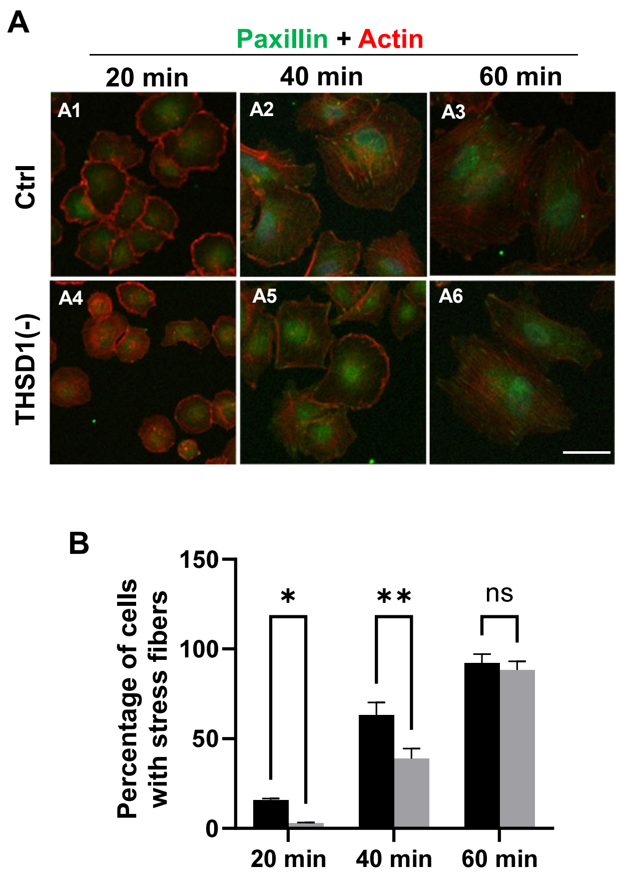

Supplemental Figure S4: Inactivation of THSD1 delays the process of cell spreading

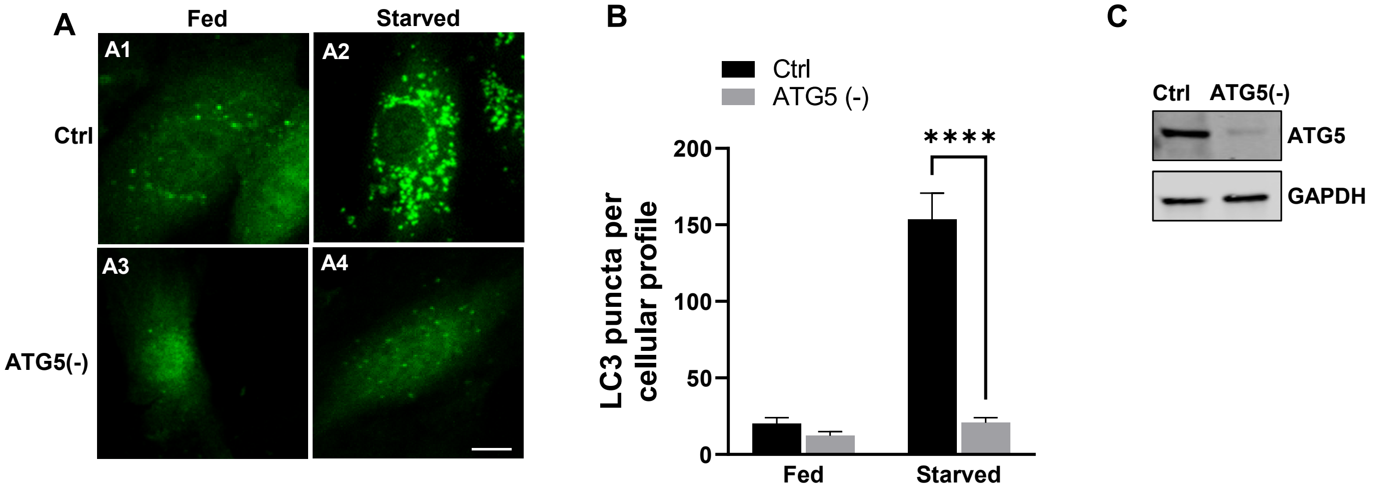

Supplemental Figure S5: Evaluation of GFP-LC3 reporter in human brain microvascular endothelial cells.

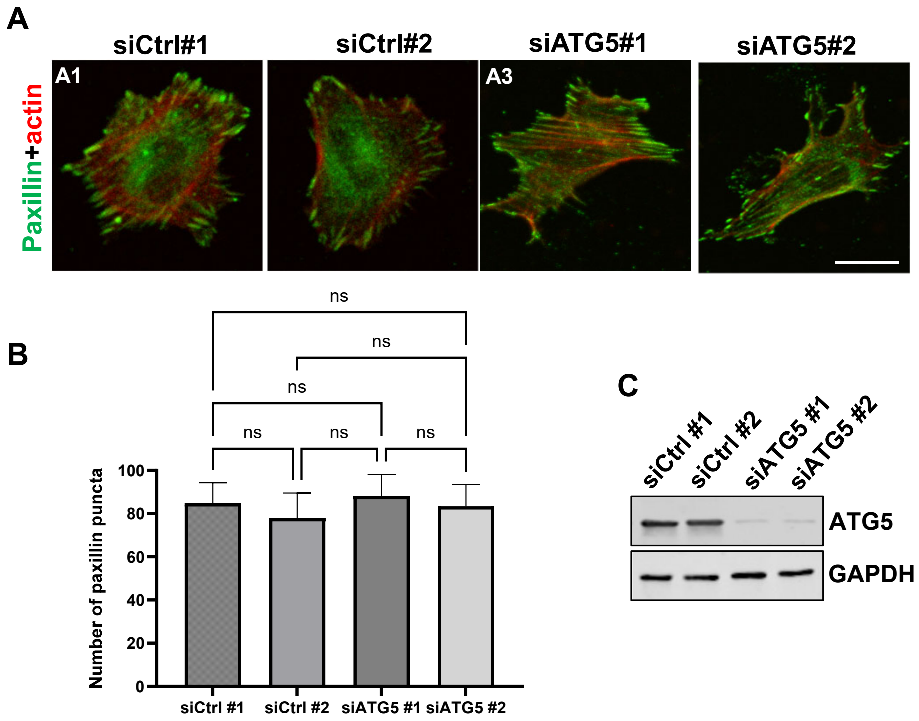

Supplemental Figure S6: Two siRNAs against ATG5 have similar effects on FA stability

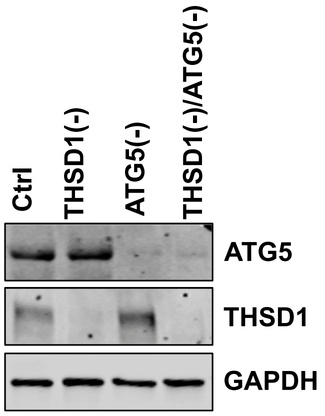

Supplemental Figure S7: Knockdown efficiency of ATG5 or THSD1 was confirmed by Western blot.

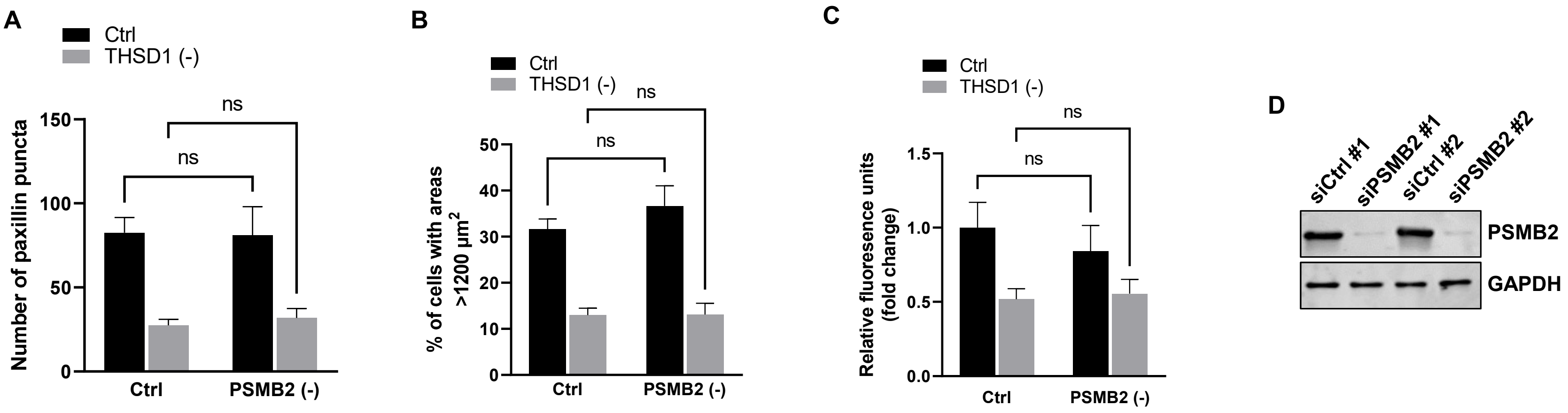

Supplemental Figure S8: Inactivation of PSMB2 has no effects on focal adhesion number, cell spreading, and attachment in HBMECs.

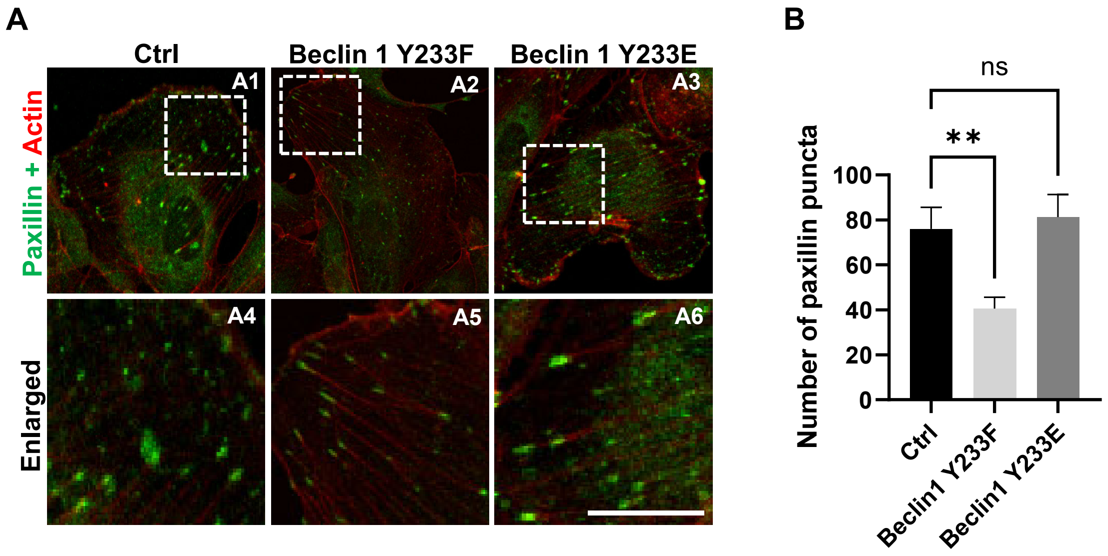

Supplemental Figure S9: Beclin 1 Y233F mutant negatively regulates focal adhesion stability

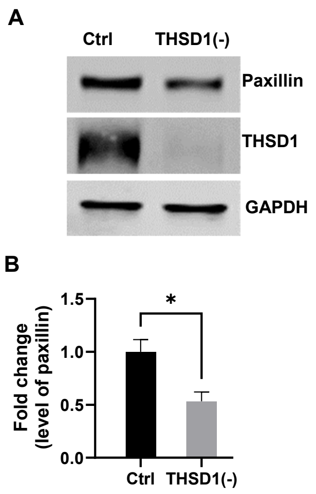

Supplemental Figure S10: Inactivation of THSD1 reduces the level of paxillin
